# The antitumor activity of CYB-L10, a human topoisomerase IB catalytic inhibitor

**DOI:** 10.1080/14756366.2018.1516651

**Published:** 2019-03-25

**Authors:** Qian Yu, Yu Chen, Hui Yang, Hong-Li Zhang, Keli Agama, Yves Pommier, Lin-Kun An

**Affiliations:** a School of Pharmaceutical Sciences, Sun Yat-sen University, Guangzhou, China;; b Developmental Therapeutics Branch and Laboratory of Molecular Pharmacology, Center for Cancer Research, National Cancer Institute, Bethesda, MD, United States

**Keywords:** Indolizinoquinolinedione, topoisomerase, DNA damage, cytotoxicity, antitumor

## Abstract

DNA topoisomerase IB (TOP1) is a validated target for discovery and development of antitumor agents. Four TOP1 poisons are clinically used for tumor treatment now. In spite of their effectiveness in solid tumors, these camptothecin (CPT) poisons suffer from many shortcomings. Therefore, many investigations have focused on the discoveries of non-CPT poisons and catalytic inhibitors. Herein, we systematically study the antitumor activity of CYB-L10, a novel indolizinoquinolinedione TOP1 catalytic inhibitor discovered in our laboratory. The results indicated that CYB-L10 mainly acts on TOP1 in cancer cells and is not a substrate of the P-glycoprotein. In addition, CYB-L10 can induce apoptosis of HCT116 cells, shows high cytotoxicity against 60 human clinical cancer cell lines (NCI60) with the mean-graph midpoint for growth inhibition of all cancer cell lines of 0.050 µM concentration and obvious antitumor efficiency *in vivo* in the HCT116 xenograft model.

## Introduction

DNA topoisomerase IB (TOP1) is an essential enzyme that controls the DNA topology structure in many cellular metabolic processes, including replication, transcription, and recombination^1–4^. TOP1 functions through a nucleophilic tyrosine residue (Tyr 732), which cleaves one phosphodiester backbone of DNA double strand, and covalently attaches to the 3′-end of the nicked DNA to form a transient enzyme-DNA covalent complex (TOP1cc)^2^^,^^5^. Inhibition of TOP1 catalytic activity or trapping of TOP1cc can result in DNA damage, which triggers apoptotic mechanisms and other cell death processes^6–8^.

TOP1 inhibitors are grouped into two types as TOP1 “poisons” and “catalytic inhibitors” based on their molecular mechanism of action. TOP1 poisons are able to trap TOP1cc to prevent further religation and thus leading to irreversible DNA strand breaks^7^^,^^9^^,^^10^. Camptothecin (CPT) and many of its derivatives are the well-known TOP1 poisons. At present, four CPT derivatives have been approved for clinical treatment of tumor, including topotecan and irinotecan approved by FDA^7^^,^^10^, belotecan (in South Korea) and 10-hydroxy camptothecin (in China)^7^^,^^11^^,^^12^. Unlike poisons, TOP1 catalytic inhibitors act at the upstream stage of the catalytic DNA cleavage reaction of enzymes, and prevent the formation of TOP1cc^13–16^.

**Figure 1.**
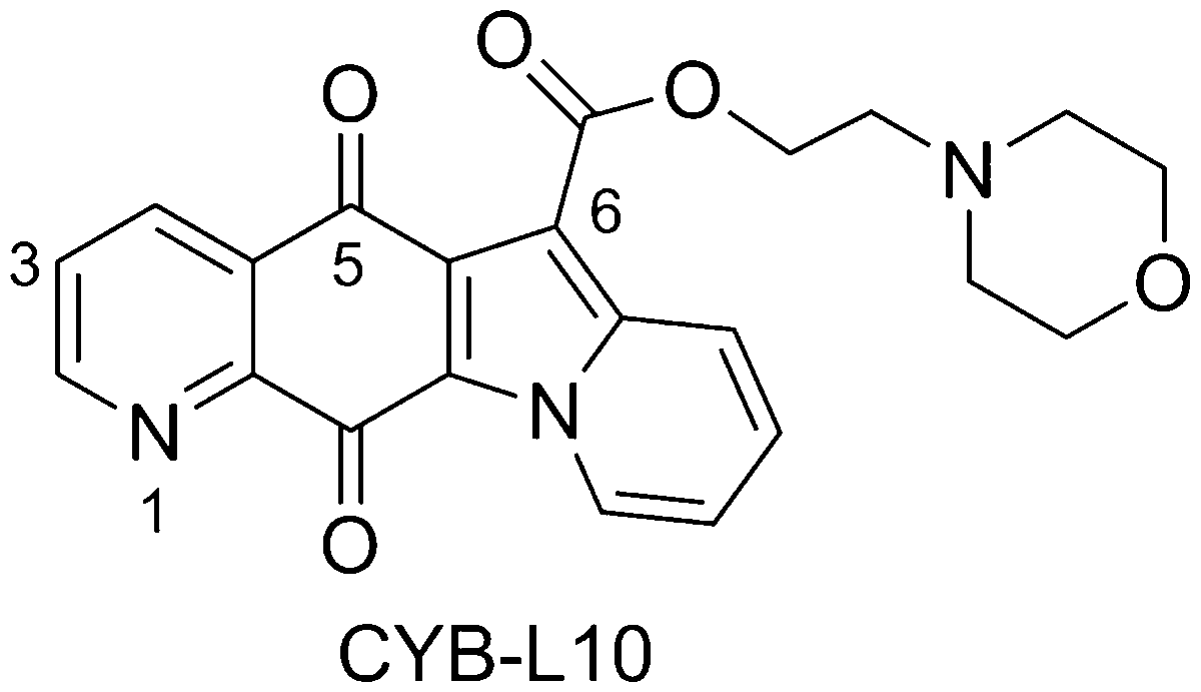
Chemical structure of CYB-L10.

In spite of their effectiveness in solid tumors, CPT poisons suffer from many shortcomings, including bone marrow dose-limiting toxicity, severe gastrointestinal toxicity^17^, poor solubility, chemically instability under physiological pH, and drug efflux-mediated resistance^10^. Therefore, many investigations have focused on the discoveries of non-CPT poisons and the catalytic inhibitors^13^^,^^14^^,^^18–21^. In our previous study, the indolizinoquinolinedione derivatives have been discovered as a new class of TOP1 catalytic inhibitors^13^^,^^15^^,^^22^, which can inhibit TOP1 catalytic cleavage reaction, and prevent the formation of TOP1cc^13^. Further structural modification led to the discovery of several TOP1 catalytic inhibitors, among which CYB-L10 (Figure 1) showed good cytotoxicity and higher TOP1 inhibition than CPT without TOP1-mediated unwinding effect^15^. Therefore, it is worthy to systematically study its antitumor activity. Herein, we report the activity of CYB-L10 *in vitro* against 60 clinical cancer cell lines and *in vivo* in HCT116 xenograft mice model.

## Methods and materials

### General experiments

The human wild-type cancer cell lines HCT116 and DU-145, the resistant cell lines HCT116-siTop1 and RC0.1 were a kind gift from Dr. Y. Pommier (Laboratory of Molecular Pharmacology, Center for Cancer Research, NCI, NIH). The human wild-type cancer cell lines MCF-7, and HepG2, and the resistant cell lines MCF-7/ADR and HepG2/ADR were a kind gift from Dr. X. Z. Bu (School of Pharmaceutical Sciences, Sun Yat-sen University). CYB-L10 (molecular weight: 405.41) was synthesized according to our reported method (compound’s code: **26**)^15^. The structure was determined by NMR and MS spectra. The purity of CYB-L10 was determined to be more than 95% through HPLC.

### Cell culture and MTT assay

The cells were cultured in RPMI-1640 or DMEM medium at 37 °C in a humidified atmosphere with 5% CO_2_. All cells to be tested in the following assays had a passage number of 3–6.

For the drug treatment experiments, the cancer cells were treated with CYB-L10 (predissolved in DMSO) at a five-dose assay concentration of 0.01, 0.1, 1, 10, and 100 µM. After incubation for 72 h at 37 °C, the MTT solution (50 µL, 1 mg/mL) in PBS (PBS without MTT as the blank) was fed to each well of the culture plate (containing 100 µL medium). After 4 h incubation, the formazan crystal formed in the well was dissolved with 100 µL of DMSO for optical density reading at 570 nm. The GI_50_ value was calculated by nonlinear regression analysis (GraphPad Prism).

### NCI60 assay

The NCI60 (National Cancer Institute 60) tumor cell drug discovery panel was developed as a tool to assess the anticancer activity of compounds against a range of cell lines derived from nine cancer cell types, including hematological malignancies, lung, central nervous system, melanoma, colorectal, renal, breast, ovarian, and prostate^18^^,^^23^^,^^24^. CYB-L10 was tested using the protocols by the NCI, which has been described previously. Briefly, CYB-L10 was tested at a five-dose assay concentration of 0.01, 0.1, 1, 10, and 100 µM for a period of 48 h. The data consist of concentration values (GI_50_) for each cell line at which the concentrations of compound that resulted in 50% cell growth inhibition. The overall antiproliferative potential is quantified as a mean-graph midpoint (MGM).

### Flow cytometry

HCT116 cells (3.0 × 10^5^ cells/mL) were grown in culture medium on 6-well plates and incubated in the presence or absence of CYB-L10 (1, 3, and 9 µM) for 24 h. And then, the cells were harvested and washed with cold PBS buffer, resuspended in 1 × binding buffer, and then stained with 5 µL Annexin V-FITC and 5 µL propidium iodide (KeyGEN BioTECH, Nanjing, China) for 15 min in dark. The stained cells were analyzed by using flow cytometry (BD, FACSCalibur, Franklin Lakes, NJ, USA) within 1 h. The experiments were repeated independently for three times.

### Pharmacokinetic study in rat

Male Sprague-Dawley rats (weighing 220–250 g, *n* = 2) were treated with CYB-L10 predissolved in 10% DMSO and 10% Kolliphor^®^ HS15 (a nonionic solubilizer) by intravenous injection (i.v., 1 mg/kg) and intragastric administration (i.g., 5 mg/kg), respectively. Blood samples (200 µL) were collected into heparinized tubes via the jugular vein at the following times: 0.083, 0.25, 0.5, 1, 2, 5, 7, and 24 h after dosing. Plasma samples (100 µL) were obtained after centrifugation for 10 min at 3000 rpm and stored at −20 °C until used for analysis. The plasma was detected through LC-MS/MS.

### *In vivo* antitumor activity

Athymic nude mice bearing the nu/nu gene were obtained from Laboratory Animal Center of Sun Yat-sen University and maintained in pathogen-free conditions to establish the model of xenografts of HCT116. All animals were used under the Policy on the Care and Use of Laboratory Animal of Sun Yat-sen University. Male nude mice 4–5 weeks old weighing 12–15 g were used. HCT116 tumor preinduced in the mice by subcutaneously injecting of HCT116 cells (100 µL, 1 × 10^7^ cells) was implanted. When the implanted tumors had reached a volume of about 80 mm^3^, the mice were randomly divided into three groups (*n* = 5) and administered by i.p. injection at a frequency of once every 3 days. The testing groups were treated with CYB-L10 at 20 mg/kg and 6.7 mg/kg dose, respectively. The control group was treated with an equivalent volume of saline. Tumor volumes (V) were monitored by caliper measurement of the length and width, and calculated using the formula: *V* = (larger diameter) × (smaller diameter)^2^/2, and growth curves were plotted using average tumor volume within each experimental group at the set time points. At the end of treatment, the animals were sacrificed, and the tumors were removed and weighed. The tumor weight inhibition (TWI) was calculated according to the formula: TWI = (1 − Mean tumor weight of the experimental group/Mean tumor weight of the control group) × 100%. Statistical comparisons were conducted using a one-way analysis of variance, followed by Tukey’s test.

## Results and discussion

### Cytotoxicity for NCI60 cell lines

CYB-L10 (Figure 1) was potent both on TOP1 inhibition and cytotoxicity^15^, but exhibit very weak inhibition against TOP2 at 25 μM (Figure S1). It was selected and submitted to National Cancer Institute (NCI, USA) for a developmental therapeutics assay against the 60 clinical tumor cell lines representing nine tissue types (NCI60)^23–25^. The tumor cells were incubated with CYB-L10 for 48 h and stained with sulphorhodamine B dye. Cell growth inhibition (GI_50_) was calculated relative to the control without compound. As shown in Table 1, CYB-L10 exhibited high cytotoxicity with a mean graph midpoint (MGM) for growth inhibition of all human cancer cell lines of 0.050 µM, and its GI_50_ values below the tested minimum concentration (10 nM) for 15 tumor cell lines.

**Table 1.** Cytotoxicity of CYB-L10 against NCI60 cell lines.

Panel	Cell line	GI_50_ (μM)^a^	Panel	Cell line	GI_50_ (μM)
	MGM^b^	0.050	Colon cancer	COLO 205	0.137
Leukemia	CCRF-CEM	0.379		HCC-2998	0.252
	HL-60 (TB)	0.24		HCT116	0.0417
	K-562	0.0282		HCT-15	0.0395
	MOLT-4	0.217		HT29	0.58
	RPMI-8226	0.166		KM12	0.168
	SR	<0.01		SW-620	<0.01
Non-small cell lung cancer	A549/ATCC	0.0205	Renal cancer	786-0	0.029
	EKVX	1.36		A498	0.0343
	HOP-62	<0.01		ACHN	<0.01
	NCI-H226	0.0289		CAKI-1	0.0339
	NCI-H23	<0.01		RXF 393	0.0351
	NCI-H322M	0.0977		SN 12C	<0.01
	NCI-H460	<0.01		TK-10	0.361
	NCI-H522	0.0119		UO-31	0.013
CNS cancer	SF-268	0.0185	Breast cancer	MCF7	<0.01
	SF-295	0.0257		MDA-MB-231/ATCC	1.62
	SF-539	<0.01		HS 578T	0.0369
	SNB-19	<0.01		BT-549	0.0558
	SNB-75	<0.01		T-47D	0.961
	U251	0.0109		MDA-MB-468	1.82
Melanoma	LOX IMVI	<0.01	Ovarian cancer	IGROV1	0.0462
	MALME-3M	0.045		OVCAR-3	0.17
	M14	<0.01		OVCAR-4	1.22
	MDA-MB-435	0.0646		OVCAR-5	0.648
	SK-MEL-2	0.0462		OVCAR-8	0.0281
	SK-MEL-28	0.0546		NCI/ADR-RES	0.0368
	SK-MEL-5	0.0285		SK-OV-3	<0.01
	UACC-257	0.0546	Prostate cancer	PC-3	0.19
	UACC-62	<0.01		DU-145	0.0305

a GI_50_ values were defined as the concentrations of compounds that resulted in 50% cell growth inhibition. The cells were incubated for 2 days with the tested compounds.

b MGM: mean graph midpoint for growth inhibition of all human cancer cell lines.

### Cytotoxicity of CYB-L10 in drug-resistant cell lines

The cytotoxicity of CYB-L10 in drug-resistant cell lines was evaluated by using the MTT assay and summarized in Table 2. HCT116-siTop1 subline was developed by transfection of colon cancer parental cells HCT116 with short hairpin RNA vectors expressing siRNA for TOP1^26^. Comparing to the parental cell line HCT116, HCT116-siTop1 subline showed 8.3-fold resistant to CPT, of which TOP1 is the only known cellular target^10^^,^^27^, and about 7-fold resistant to CYB-L10, implying that TOP1 may be the major cellular target of CYB-L10.

**Table 2.** Cytotoxicity of the CYB-L10 in drug-resistant human cancer cell lines.

Cpd.	GI_50_ ± SD (μM)^a^		Resistance ratio^b^
	Parental cell line	Resistant subline	
	HCT116	HCT116-siTop1	
CYB-L10	0.027 ± 0.024	0.19 ± 0.092	7.0
CPT	0.009 ± 0.001	0.075 ± 0.014	8.3
	DU-145	RC0.1	
CYB-L10	0.016 ± 0.010	0.65 ± 0.010	40.6
CPT	0.019 ± 0.009	7.53 ± 1.88	396.3
	MCF-7	MCF-7/ADR	
CYB-L10	0.091 ± 1.33	0.60 ± 0.21	6.6
DOX	0.15 ± 0.003	11.67 ± 1.94	77.8
	HepG2	HepG2/ADR	
CYB-L10	0.065 ± 0.030	0.74 ± 0.24	11.4
DOX	0.19 ± 0.048	9.04 ± 0.14	47.6

a GI_50_ values (means ± SD) were defined as the concentrations of compounds that resulted in 50% cell growth inhibition, and obtained from MTT assay. Every experiment was repeated at least three times.

b Resistance ratio was calculated by dividing the GI_50_ of the mutant cell line by the GI_50_ of the corresponding parental cell line.

The prostate cancer cell RC0.1 has an R364H mutation in the TOP1 relating to the wild-type parental cell DU-145^28^. The TOP1 with R364H mutation is catalytically active, but lead to RC0.1 cells resistant to CPT because the R364 residue is close to the catalytic tyrosine and can stabilize the open form of TOP1cc^29^^,^^30^. The RC0.1 cells were highly resistant to TOP1 poison CPT (396.3-fold) and less resistant to CYB-L10 (40.6-fold), implying that the binding site of CYB-L10 on TOP1 is different from that of CPT.

P-glycoprotein (Pgp) mediated drug efflux is generally responsible for classical multiple drug resistance^31^. The chemotherapeutic agent doxorubicin (DOX) is a substrate of Pgp, and has been found highly resistant for breast cancer MCF-7/ADR (77.8-fold) and hepatocellular HepG2/ADR sublines (47.6-fold), both with overexpressed Pgp^32^. However, CYB-L10 appeared not to be a substrate of the Pgp (Table 2).

### Apoptosis analysis of CYB-L10

**Figure 2.**
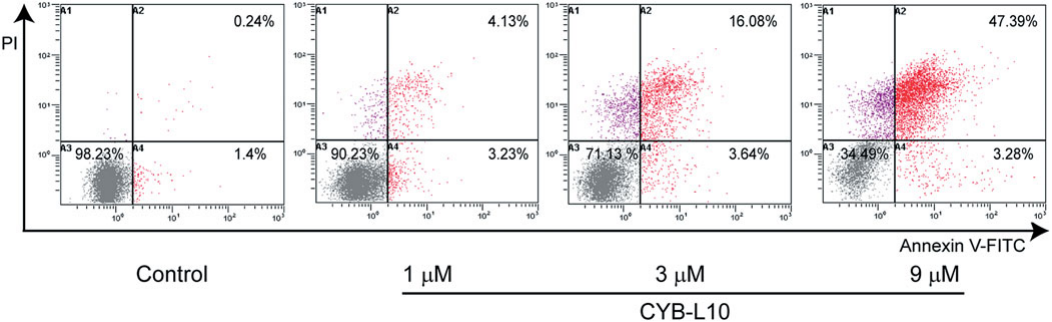
The flow cytometry histograms analysis. HCT116 cells were treated with CYB-L10 at 1, 3, and 9 μM, respectively for 24 h (*n* = 3).

To estimate the effect of CYB-L10 on apoptosis, flow cytometry analysis using double staining with *annexin V-FITC/PI* was carried out for HCT116 cells. As shown in Figure 2, after being treated with CYB-L10 (1, 3, and 9 μM) for 24 h, compared to the untreated control group, the apoptotic cells in the treated group showed an increase in a dose-dependent manner. CYB-L10 induced the major population of HCT116 cells into the late apoptotic stage 47.39% at 9 μM concentration.

### Pharmacokinetic study of CYB-L10 *in vivo*

The *in vivo* pharmacokinetic (PK) study of CYB-L10 was conducted in SD rats. The SD rats were divided into two groups (*n* = 2) and treated by i.v. injection at 1 mg/kg dose and i.g. at 5 mg/kg dose, respectively. The plasma samples were collected up to 24 h postdose and measured for the concentration of CYB-L10. The PK parameters were calculated and summarized in Table 3. By i.v. administration, the AUC_0→∞_ was 241 ± 40 h·ng/mL, and *T_1/2_* was 18.4 ± 5.12 h. After oral administration, the *T_max_*, *C_max_* and AUC_0→∞_ were 0.46 ± 0.10 h, 24.7 ± 6.59 ng/mL and 76.0 ± 16.5 h·ng/mL, respectively. The bioavailability (F) was 6.3%.

**Table 3.** PK parameter of CYB-L10.

Parameters	Mean ± SD	
	i.v. (1 mg/kg)	i.g. (5 mg/kg)
*T_max_* (h)	–	0.46 ± 0.10
*C_max_* (ng/ml)	–	24.7 ± 6.59
AUC_0→∞_ (h⋅ng/ml)	241 ± 40	76.0 ± 16.5
*T_1/2_* (h)	18.4 ± 5.12	18.4 ± 7.77
*F* (%)	–	6.3

### Antitumor activity *in vivo* of CYB-L10

**Figure 3.**
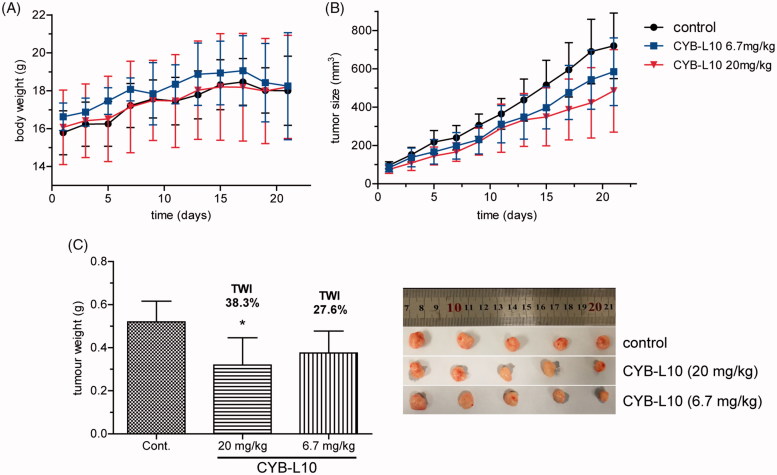
The antitumor efficiency of CYB-L10 *in vivo*. The effects of CYB-L10 on body weight (A), tumor size (B), and tumor weight (C) at the dose of 6.7 mg/kg and 20 mg/kg in the HCT116 xenograft model. Statistically significant difference in mean tumor weight compared with the control, *p < 0.05.

A human colon cancer HCT116 xenograft nude mice model was established to evaluate the antitumor efficiency of CYB-L10 *in vivo*. Mice were randomly divided into three groups and administered i.p. injection at a frequency of once every 3 days. Two groups were treated with CYB-L10 at 20 mg/kg and 6.7 mg/kg dose, respectively. The negative control group was treated with an equivalent volume of saline. As shown in Figure 3(B), administration of CYB-L10 obviously reduced the tumor volume of the nude mice in a dose-dependent manner comparing to the negative control group. Finally, the tumor weight inhibitions (TWI) of CYB-L10 administration groups were 38.3% (20 mg/kg, *p* < 0.05) and 27.6% (6.7 mg/kg), respectively (Figure 3(C)). Meanwhile, the weight of the body of the mice in the CYB-L10 administration groups had no obvious loss comparing to the negative control group (Figure 3(A)).

## Conclusion

Indolizinoquinolinedione derivative CYB-L10 serves as a novel TOP1 catalytic inhibitor with high cytotoxicity and higher TOP1 inhibition than that of CPT. Further study indicated that CYB-L10 exhibits high cytotoxicity against 60 clinical cancer cell lines from NCI (NCI60) with MGM value of 0.050 µM and induce apoptosis of HCT116 cells in the late apoptotic stage, implying CYB-L10 is a good anticancer candidate. The drug-resistant cell assays indicated that CYB-L10 may mainly act to TOP1 in tumor cells and is not a substrate of the Pgp, a drug efflux protein involving in multiple drug resistance. CYB-L10 was also evaluated on PK and antitumor efficiency *in vivo*, and found to have long *T_1/2_* value and obvious antitumor efficiency in HCT116 xenograft nude mice model with TWI of 38.3% at 20 mg/kg dose.

## Supplementary Material

Supplemental_material.pdf
