# Supplementary material for: The antitumor activity of CYB-L10, a human topoisomerase IB catalytic inhibitor
Source: J Enzyme Inhib Med Chem. 2019 Mar 25;34(1):818–22. doi: 10.1080/14756366.2018.1516651 (PMC6442119; doi:10.1080/14756366.2018.1516651)
Supplement: Supplemental_material.pdf [file IENZ_A_1516651_SM0316.pdf]

SUPPLEMENTARY DATA FOR

The antitumor activity of CYB-L10, a human topoisomerase IB catalytic inhibitor

Qian Yu[a], Yu Chen[a], Hui Yang[a], Hong-Li Zhang[a], Keli Agama[b], Yves Pommier[b], Lin-Kun An[a,*]

[a]School of Pharmaceutical Sciences, Sun Yat-sen University, Guangzhou, 510006, China; [b]Developmental Therapeutics Branch and Laboratory of Molecular Pharmacology, Center for Cancer Research, National Cancer Institute, Bethesda, MD 20892-4255, United States

*: Corresponding author: Lin-Kun An

Tel. and fax: +86-20-39943413

E-mail address: lssalk@mail.sysu.edu.cn

Content

Figure S1(TOP2-mediated relaxation assay) ……………………………………………………..…S2

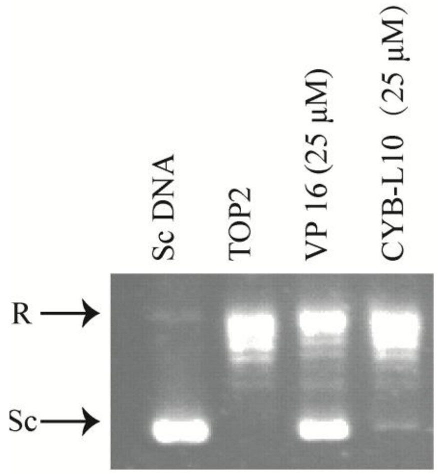

Figure S1. TOP2-mediated relaxation assay. Lane 1, supercoiled pBR322 DNA alone; Lane 2, DNA and enzyme; Lanes 3, DNA, enzyme and the positive control etoposide (VP16) at 25 μM; Lanes 4, DNA, enzyme and CYB-L10 at 25 μM. R: relaxed DNA. Sc: supercoiled DNA.
